# Isolated Anterior Lens Capsule Rupture Secondary to Blunt Trauma: Pathophysiology and Treatment

**DOI:** 10.4274/tjo.85547

**Published:** 2016-08-15

**Authors:** Mehmet Serhat Mangan, Ceyhun Arıcı, İbrahim Tuncer, Hüseyin Yetik

**Affiliations:** 1 Okmeydanı Training and Research Hospital, Ophthalmology Clinic, İstanbul, Turkey; 2 İstanbul University Cerrahpaşa Faculty of Medicine, Department of Ophthalmology, İstanbul, Turkey; 3 Alfagöz Medical Center, İzmir, Turkey

**Keywords:** Blunt eye trauma, lens anterior capsule rupture, traumatic cataract

## Abstract

A 25-year-old man suffered an isolated lens anterior capsular tear and mature cataract formation following blunt injury to his right eye. One week after the trauma, best-corrected visual acuity (BCVA) in the right eye was hand motion. B-scan ultrasonography showed that the lens posterior capsule was intact; no vitreous foreign body or retinal pathology were observed. Orbital computed tomography revealed narrowed anterior chamber and increased lens material volume and lens reflectivity in the injured right eye. The globe was intact and no bone fractures were observed. The cataractous lens material was removed by phacoemulsification and a foldable, acrylic, posterior chamber intraocular lens was implanted in the bag. Postoperative BCVA in the right eye was 20/20.

## INTRODUCTION

Isolated anterior lens capsule tears due to blunt ocular trauma are rare.^1,2,3,4,5^ In this report we aimed to describe the clinical findings, mechanism of development, and surgical treatment applied in the case of a patient with isolated anterior lens capsule rupture due to blunt trauma with a wooden object.

## CASE REPORT

**Figure 1:**
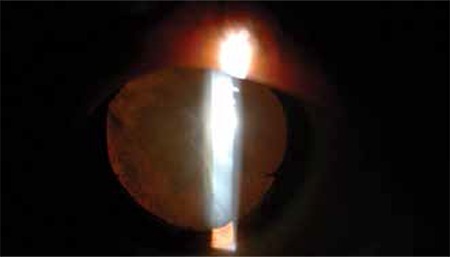
Biomicroscopic anterior segment photograph of the isolated anterior lens capsule extending from 7 to 11 o’clock

A 25-year-old male presented with periocular pain the day after sustaining a blunt trauma to the right eye with a piece of wood. At presentation, his best corrected visual acuity (BCVA) was 1/10 in the right eye and 10/10 in the left eye. Intraocular pressure (IOP) was 10 mmHg in the right and 12 mmHg in the left eye. Slit-lamp examination of the right eye revealed mild eyelid edema, 1+ conjunctival injection, clear cornea, an anterior lens capsule rupture bissecting the lens capsule along the 7 to 11 o’clock line extending under the superior and inferior iris margins to the lens equator, and traumatic cataract that did not yet seriously impede visualization of the fundus (Figure 1). Retinal examination done at this stage revealed no pathology in the posterior pole, midperipheral or peripheral retina.

**Figure 2:**
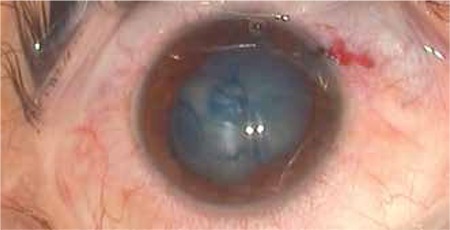
Intraoperative image of the severe traumatic cataract and the anterior lens capsule rupture visualized with trypan blue

**Figure 3:**
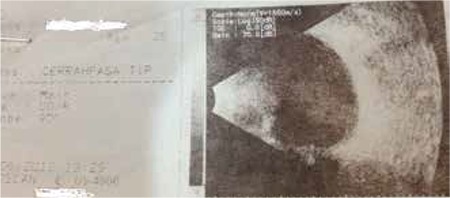
Preoperative B-scan ultrasonography image

**Figure 4:**
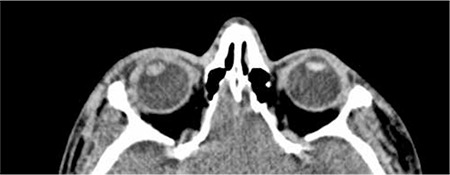
Preoperative orbital computed tomography image

Visual acuity was 1/10 on the third day after the trauma; however, due to increasing lens opacity, it decreased to hand motions by day 7 (Figure 2). B-scan ultrasonography and orbital computed tomography (CT) were performed to rule out foreign body and globe rupture and to document the patient’s current status. No intravitreal foreign body or retinal pathology was detected on B-scan ultrasonography (Figure 3). Orbital CT revealed narrowed anterior chamber and increased lens volume and reflectivity compared to the healthy eye. Furthermore, the globe was observed to be intact and no fractures were detected in the bones of the orbit (Figure 4).

The patient’s trauma was graded using the Birmingham Eye Trauma Terminology (BETT) and Ocular Trauma Score (OTS). BETT grade C was determined based on the injury being closed globe and contusional as well as the patient’s visual acuity at presentation. No relative afferent pupillary defect was observed. The extent of the injury was zone II. The patient’s OTS raw points value was 90, corresponding to an OTS of 4.

**Figure 5:**
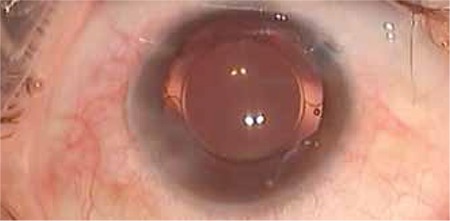
Postoperative anterior segment image

Surgery was planned to restore the visual acuity lost due to progressive traumatic cataract. The procedure consisted of entering the anterior chamber via a 2.8 mm clear cornea incision made temporally with 20 gauge MVR blades, trypan blue capsule staining under air and lens extraction by the bimanual irrigation/aspiration technique. A foldable, acrylic posterior capsule intraocular lens was implanted by injection into the sac and anterior capsulorhexis was performed in half-circles on the temporal and nasal sides in the pupillary plane. The zonules and posterior capsule were observed as intact after the procedure. The corneal incisions were closed using hydration, without sutures (Figure 5). Uncorrected visual acuity was 20/20 on the first postoperative day.

## DISCUSSION

Crystalline lens damage secondary to blunt ocular trauma can result in lens dislocation, subluxation or posterior capsule rupture.^6,7,8^

Posterior capsule rupture occurs more frequently than anterior capsule rupture.^6,7,8^ Gampanella et al.^9^ stated that the mechanism of posterior capsule rupture due to blunt trauma involves the anatomic relationship between the vitreous and the lens interface. According to this theory, the Wiegert ligament, which connects the anterior cortical vitreous and the posterior lens capsule, usually attaches in the midperipheral region of the lens. This connection weakens with age. Secondary to the rapid compression of the eye on the anterior-posterior axis and the expansion that immediately follows, the Wiegert ligament causes the posterior lens capsule to tear.

According to a hypothesis from Banitt et al.,^3^ isolated anterior lens capsule tear secondary to blunt trauma probably occurs as a result of the rapid focal indentation of the cornea onto the lens (coup injury) or a rebound effect secondary to the trauma in which the vitreous applies high pressure to the lens (countrecoup injury).^3^

We believe that with less severe injuries, expansion following ocular compression on the anterior-posterior axis leads to posterior capsule tear, while in more severe injuries the anterior-posterior axis compression may cause anterior capsule rupture before the subsequent expansion. Especially in young patients like the current case in which the anterior hyaloid is tight, the vitreous compact, and the zonules intact, the anterior capsule rupture is limited to the equatorial plane and does not continue to the posterior capsule. The tight anterior hyaloid likely buffers the force of the direct impact but transfers the energy toward the lens due to the countrecoup effect. Therefore, although the retina and posterior pole are protected from trauma, the impact results in anterior capsule rupture because of the elasticity of the lens material. In cases like this, in which the patient is young and the tissues are resilient, the damage is limited and cataractous lens material can be removed by irrigation-aspiration alone, resulting in favorable anatomic and visual outcomes.

### Ethics

Informed Consent: It was taken.

Peer-review: Externally peer-reviewed.
